# Overexpression of *abscisic acid-insensitive gene ABI4* from *Medicago truncatula*, which could interact with ABA2, improved plant cold tolerance mediated by ABA signaling

**DOI:** 10.3389/fpls.2022.982715

**Published:** 2022-09-23

**Authors:** Yinruizhi Li, Mengdi Wang, Tao Guo, Shuwen Li, Ke Teng, Di Dong, Zhuocheng Liu, Chenyan Jia, Yuehui Chao, Liebao Han

**Affiliations:** ^1^Turfgrass Research Institute, College of Grassland Science, Beijing Forestry University, Beijing, China; ^2^Chongqing Key Laboratory of Germplasm Innovation and Utilization of Native Plants, Chongqing Landscape and Gardening Research Institute, Chongqing, China; ^3^Beijing Research and Development Center for Grass and Environment, Beijing Academy of Agriculture and Forestry Sciences, Beijing, China; ^4^Inner Mongolia Mengcao Ecological Environment (Group) Co., Ltd., Hohhot, China

**Keywords:** *ABI4*, *Medicago truncatula*, cold tolerance, xanthoxin dehydrogenase, ABA

## Abstract

ABI4 is considered an important transcription factor with multiple regulatory functions involved in many biological events. However, its role in abiotic stresses, especially low-temperature-induced stress, is poorly understood. In this study, the *MtABI4* gene was derived from *M. truncatula*, a widely used forage grass. Analysis of subcellular localization indicated that ABI4 was localized in the nucleus. Identification of expression characteristics showed that *ABI4* was involved in the regulatory mechanisms of multiple hormones and could be induced by the low temperature. IP-MS assay revealed that MtABI4 protein could interact with xanthoxin dehydrogenase protein (ABA2). The two-hybrid yeast assay and the biomolecular fluorescence complementarity assay further supported this finding. Expression analysis demonstrated that overexpression of *MtABI4* induced an increase in *ABA2* gene expression both in *M. truncatula* and *Arabidopsis*, which in turn increased the ABA level in transgenic plants. In addition, the transgenic lines with the overexpression of *MtABI4* exhibited enhanced tolerance to low temperature, including lower malondialdehyde content, electrical conductivity, and cell membrane permeability, compared with the wide-type lines after being cultivated for 5 days in 4°C. Gene expression and enzyme activities of the antioxidant system assay revealed the increased activities of SOD, CAT, MDHAR, and GR, and higher ASA/DHA ratio and GSH/GSSG ratio in transgenic lines. Additionally, overexpression of *ABI4* also induced the expression of members of the Inducer of CBF expression genes (ICEs)-C-repeat binding transcription factor genes(CBFs)-Cold regulated genes (CORs) low-temperature response module. In summary, under low-temperature conditions, overexpression of *ABI4* could enhance the content of endogenous ABA in plants through interactions with ABA2, which in turn reduced low-temperature damage in plants. This provides a new perspective for further understanding the molecular regulatory mechanism of plant response to low temperature and the improvement of plant cold tolerance.

## Introduction

*abi4 abscisic acid-insensitive 4* was originally named after a series of ABA-insensitive mutants identified through a forward genetic screen (Finkelstein, 1994). In recent years, *ABI4* has been recognized as a core transcription factor with multiple regulatory functions, which is involved in many important biological events. For example, *ABI4* was associated with seed germination and seedling morphogenesis. The expression of *ABI4* gradually decreased with seed germination and seedling morphology (Shkolnik-Inbar and Bar-Zvi, 2011). Accordingly, overexpression of *ABI4* exacerbated the ABA-induced inhibition of seed germination in *Arabidopsis* (Finkelstein et al., 2011). *ABI4* also played some significant function in plastid/mitochondrial reverse signaling, especially acting as a downstream target of the *GUN1* in mediating the PGE pathway regulating plastid (Sun et al., 2011). Moreover, ABI4 was also involved in sugar signaling. Some studies showed that *ABI4* regulates the expression of the sugar-responsive gene by binding to CE1-like motif elements in the promoter region, such as the *ADP-glucose pyrophosphorylase large subunit ApL3* in the starch synthesis pathway and *precipitate branching enzyme SBE2.2* in the starch synthesis pathway (Bossi et al., 2009). *ABI4* affects lateral root production. *abi4* mutants increase lateral root density and length compared to the wild type, while their overexpression reduced lateral root production (Shkolnik-Inbar and Bar-Zvi, 2010).

Additionally, reactive oxygen species (ROS) are also involved in the ABI4-mediated reverse signaling pathway (León et al., 2012). Overall, ABI4 plays an integral role in plant growth, development, and response to adversity. Although in recent years studies have led to a certain understanding of the function of ABI4, its role remains incomplete, such as its regulatory mechanism in the low-temperature response.

Plant cold tolerance is a complex trait controlled by multiple genes, such as *COR*, *ICE*, *CBF*, and *GPAT*, which all play an important role in plant response to low-temperature stress and in improving cold tolerance (Zhu et al., 2019; Tang et al., 2020). In plants, the most widely studied low-temperature response signaling pathway is the CBF/DREB1 signaling pathway with the main upstream and downstream modules Inducer of CBF expression genes (ICEs)-C-repeat binding transcription factor genes (CBF)/DREB1s-Cold regulated genes (CORs). ICE1 binds to the promoter region of the *CBF* gene to activate the expression, thereby mediating cold tolerance in plants (Chinnusamy et al., 2007). Low-temperature signals do not directly regulate *ICE1* gene transcription, but the *E3 ligase HOS1* (*high osmotic expression1*) is involved in ICE1 ubiquitination and degradation via the 26S protease pathway, thereby affecting ICE1 protein stability (Shi et al., 2015). The activity of OST1 kinase (*open stomata1*) was found to be rapidly induced by low temperature, and ICE1 and activated OST1 interacted to phosphorylate ICE1 protein and improve its binding capacity to the *CBF3* promoter region and ICE1 stability (Ding et al., 2019), and it was also found that OST1 phosphorylated BTF3L and BTF3 and promoted their interaction with CBF (Ding et al., 2018). The Jas domains of JAZ1 and JAZ4 interacted with the C-terminal of the ICE1 protein and inhibited ICE1 activity in *A. mimosa*. When exposed to low-temperature stress, JAZ proteins were degraded by increased JA content activating JA signaling, thereby relieving the inhibitory effect of ICE1 by JAZ and improving low-temperature tolerance (Hu et al., 2013).

Low temperature triggers the change in the activity and content of hormones in plants, which in turn influence the metabolic processes of plant growth. Abscisic acid (ABA) as a natural hormone defending against the abiotic stresses, plays an important role in stresses such as low temperatures (Jiang and Zhang, 2002). There is a significant increase in ABA content in leaves of *Arabidopsis* and rice at low temperatures (Eremina et al., 2016). In addition, ABA reduces the water content of plants and promotes the accumulation of soluble sugars and total phenolic content, thus improving the cold resistance of plants (Yang et al., 2016). The first hormone found to be associated with cold resistance was gibberellin (GA), and studies have found that the expression of *CRT/DRE* transcription-related genes is associated with GAs. For example, studies on the relationship between *CBF1* and gibberellin have revealed that constitutive expression of *CBF1* reduces gibberellin activity (Chen et al., 2007). Salicylic acid (SA) content increases significantly under low-temperature conditions and is detrimental to plant growth if the xconcentration of SA is too high, while low concentrations of SA promote cell expansion and growth (Klessig et al., 2018). Jasmonic acid (JA), a very important plant signaling molecule, can mediate the expression of those resistance genes, and JA interacts with CBF to regulate downstream cold-responsive genes, thereby enhancing cold tolerance in plants (Hu et al., 2017). Additionally, the increased JA content improves CAT, SOD, and APX activities, thereby enhancing plant cold tolerance (Campos et al., 2019).

There are fewer reports on the function of *ABI4* in the low-temperature response. Only one recent study was reviewed, showing that MdABI4 crosstalked jasmonic and abscisic acid signaling to regulate cold tolerance in apples (An et al., 2022). On the other hand, as a model plant of the legume family (Stacey et al., 2006), it is of great importance to investigate the low-temperature stress response genes of *M. truncatula* for exploring its cold tolerance mechanism and improving the cold tolerance ability, in agricultural production. It also has important implications for the study of other legumes. Therefore, in this study, the function and regulatory mechanisms of *MtABI4* in response to low temperature was characterized, aiming to lay the foundation for rapid breeding of cold-tolerance forage varieties of *M. truncatula* by means of genetic engineering.

## Results

### Molecular characterization and subcellular localization of MtABI4

**FIGURE 1:**
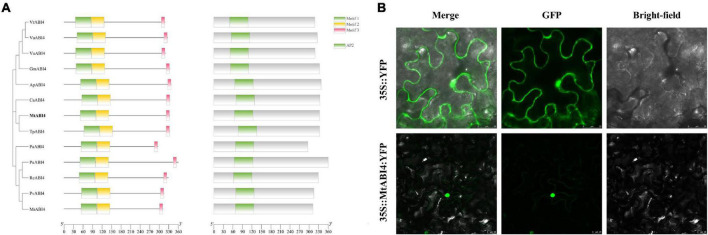
Phylogenetic tree, motif analysis, protein structural domains, and the subcellular localization of ABI4. In **(A)** different color blocks represent different motifs and structural domains. The scale bar represents the length of the amino acid sequence. **(B)** Represents the subcellular localization of ABI4.

Sequence analysis indicated that *MtABI4* possessed a complete coding region of 1,011 bp, encoding a polypeptide chain containing 336 amino acids with a predicted molecular weight of 37.8 kD. MtABI4 protein contained an AP2 DNA-binding domain, belonging to the AP2 superfamily (Supplementary Figures 1A,B). Homology and phylogenetic analysis showed that MtABI4 was more closely related to ABI4 from *Trifolium pratense*, and ABI4 with high homology from ten species all had three motifs and an AP2 domain (Figure 1A). SignalP predicted that MtABI4 had no signal peptide, and subcellular localization prediction showed that MtABI4 was an extracellular protein (Supplementary Figures 1C,D).

By observing the expression of an ABI4-GFP fusion protein in tobacco leaves, we found that ABI4 was localized in the nucleus, which was inconsistent with our previous prediction (Figure 1B).

### *MtABI4* gene responds to multiple hormones

**FIGURE 2:**
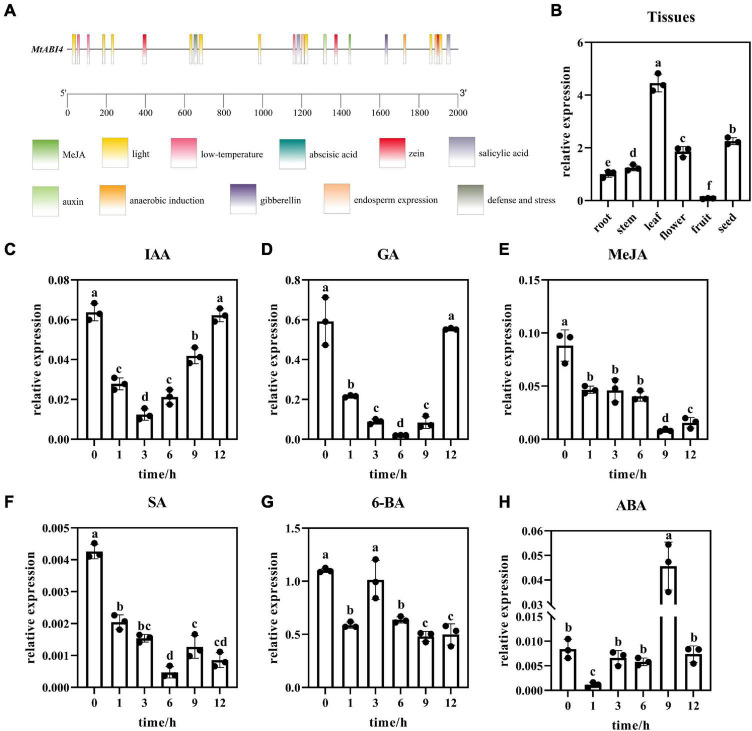
Cis-acting elements analysis and the expression patterns of *MtABI4* by qRT-PCR analysis. In graph panel **(A)**, different color blocks represent different cis-acting elements. The scale bar represents the length of the base sequence. Panel **(B)** represents the expression of *ABI4* in different tissues. Different letters in graphs indicate significant differences at *p* ≤ 0.05. Panels **(C–H)** represents the expression of *ABI4* at different time points in response to IAA, GA, MeJA, SA, 6-BA, and ABA, respectively. The values are means ± SD (*n* = 3).

To analyze the regulation mechanism of *MtABI4*, the upstream sequence of the gene, which was 2,000 bp in length, was obtained. Cis-acting elements analysis showed that the motifs enriched in the promoter region of *MtABI4* were widely involved in the abscisic acid responsiveness, anaerobic induction, auxin responsiveness, the MeJA-responsiveness, endosperm expression, low-temperature responsiveness, zein metabolism regulation, gibberellin-responsiveness, defense and stress responsiveness, salicylic acid responsiveness, and light response (Figure 2A), indicating that this gene may play a multifunctional role in response to multiple hormones and stress.

Based on this, wild-type plants were sprayed with the corresponding hormones and plant tissues were collected at different time points for analysis of the *MtABI4* gene response to these hormones. Quantitative fluorescence analysis indicated that the expression of *ABI4* was down-regulated under the treatment of IAA, 6-BA, GA, MEJA, and SA (Figures 2C–G). The difference was that *ABI4* had a rebound at 6 h under the treatment of 6-BA, and returned to the initial level at 12 h under the treatment of GA and IAA. In contrast to them, the expression level of *ABI4* showed an increasing trend under ABA treatment, with a down-regulation at 1 h and a peak at 9 h (Figure 2H). This would suggest that *ABI4* is indeed involved in the regulatory mechanisms of these hormones. In addition, tissue-specific expression analysis showed that the ABI4 gene possessed the highest expression in leaves, followed by fruits, and the lowest expression in seeds (Figure 2B).

### *MtABI4* improved the cold tolerance of the over-expressed transgenic plants

**FIGURE 3:**
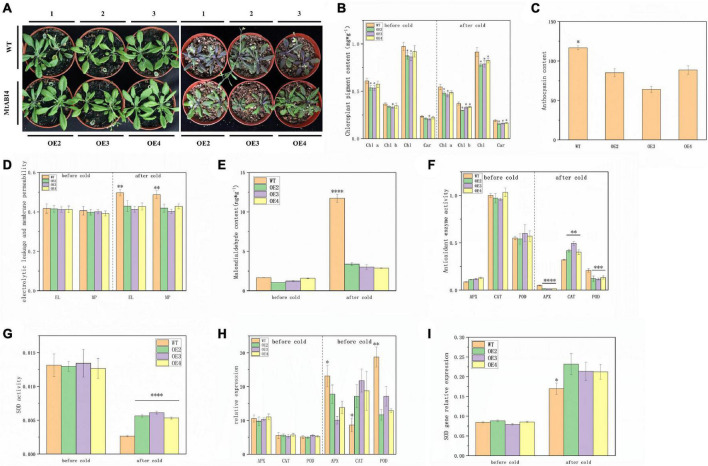
Plant growth status, physiological characteristics, and the expression of antioxidant enzyme genes at low temperatures. Panel **(A)** represents the growth condition of wild-type and transgenic plants before the low-temperature treatment and after the 5-day low-temperature treatment. Panels **(B–E)** represents the content of chloroplast pigments, anthocyanin, electrolytic leakage, membrane permeability, and malondialdehyde. Panels **(F,G)** represent the activity of APX, CAT, POD, and SOD. Panels **(H,I)** represent the expression of these antioxidant enzyme genes. The values are means ± SD (*n* = 3). *, in graphs, indicates significant differences in the means at *p* < 0.05 between each level in transgenic lines and WT plants, and ^**^, ^***^, and ^****^, in graphs, indicate significant differences in the means at *p* < 0.01, *p* < 0.001, and *p* < 0.0001, respectively.

Considering that the promoter region of the *ABI4* gene contains a low-temperature element, the transgenic lines, and the wild type was incubated at 4°C for 5 days to investigate the role of the *ABI4* gene in the low-temperature response. Phenotypic observations showed that the plants all exhibited some degree of wilting, with the wild-type plants showing darker leaf color after low-temperature treatment (Figure 3A). The results of chloroplast pigment content measurements showed that chlorophyll a, chlorophyll b, chlorophyll, and carotenoids were slightly lower in the transgenic lines than in WT, but there were no significant changes in chloroplast pigment content in the individual lines, before and after the low-temperature treatment (Figure 3B). WT exhibited higher anthocyanin content than the transgenic strain after low-temperature treatment (Figure 3C). The wild-type and transgenic lines did not show significant differences in electrolytic leakage, membrane permeability, and malondialdehyde content before the low-temperature treatment, however, these indicators in WT showed a significant increase and were much greater than in the transgenic lines, after the low-temperature treatment (Figures 3D,E). These results suggested that the transgenic plants accumulated less anthocyanin and suffered less cell membrane damage under low-temperature conditions.

Analysis of antioxidant enzyme activities showed that there were no significant differences in APX, CAT, POD, and SOD contents between wild-type and transgenic lines before low-temperature treatment. These antioxidant enzyme activities decreased after low-temperature treatment, in both WT and transgenic lines. Among them, APX and POD activities were significantly lower in the transgenic lines than in the wild type, but the opposite was true for CAT and SOD (Figures 3F,G). The expression of the antioxidant enzyme genes also showed a similar profile to the enzyme activity, the only difference being that the expression of these genes showed an overall upregulation after low-temperature treatment (Figures 3H,I). These results suggested that overexpression of the ABI4 gene under low-temperature conditions affected gene expression and enzyme activity of these antioxidant enzymes.

**FIGURE 4:**
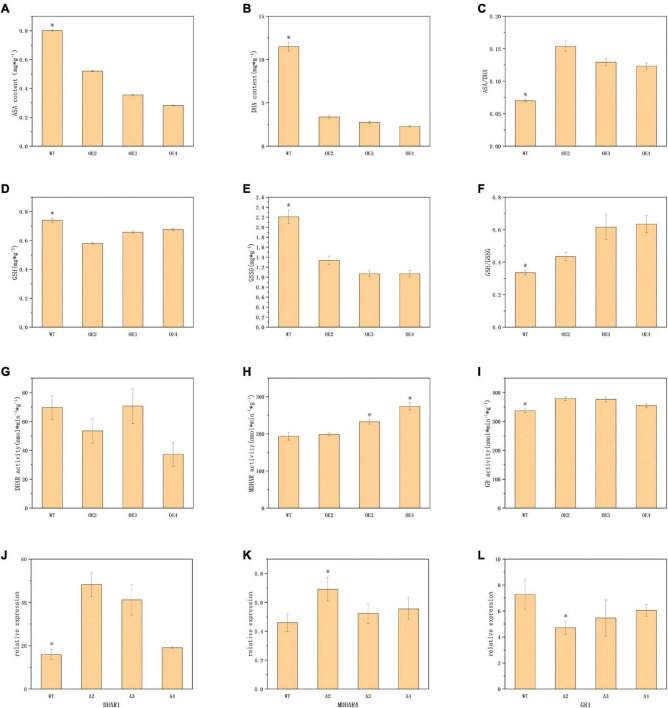
Determination of indicators related to non-enzymatic antioxidant systems. Panels **(A,B,D,E)** represents the content of ASA, DHA, GSH, and GSSG in turn. Panel **(C,F)** represents the ASA/DHA and GSH/GSSG ratio, respectively. Panels **(G–I)** represents the activities of DHAR, MDHAR, and GR. Panels **(J–L)** represents the relative expression of *DHAR1*, *MDHAR4*, and *GR1*. The values are means ± SD (*n* = 3). *, in graphs, indicates significant differences in the means at *p* < 0.05 between each level in transgenic lines and WT plants.

As a major part of the non-enzymatic antioxidant system, the ASA-GSH cycle played an important role in the scavenging of reactive oxygen species and resistance to stresses in plants (Nahar et al., 2015). Corresponding compound content analysis indicated that the transgenic plants contained lower ASA and DHA, but higher ASA/DHA (Figures 4A–C). GSH, GSSG, and GSH/GSSG also exhibited similar trends (Figures 4D–F). This would suggest that there are lower levels of oxidation in transgenic plants. Meanwhile, the activities of MDHAR and GR in transgenic lines demonstrated a significant enhancement over those in WT, while the activity of DHAR showed no significant difference (Figures 4G–I). The expression of the *DHAR1* in transgenic plants was significantly upregulated compared to the WT, but there were no significant differences in MDHAR4 and GR1 (Figures 4J–L).

### *MtABI4* can affect the expression of the Inducer of CBF expression genes-C-repeat binding transcription factor genes-cold regulated genes module and some other low-temperature responsive genes

**FIGURE 5:**
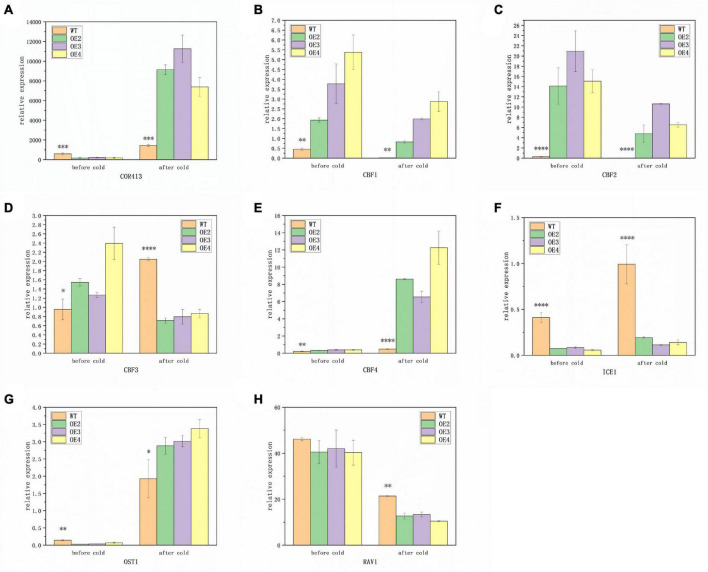
Expression patterns of genes responsive to low temperatures. **(A–H)** Represent the expression levels of *COR413*, *CBF1-4*, *ICE1*, *OST1*, and *RAV1*, in turn, under low temperatures. The values are means ± SD (*n* = 3). *, in graphs, indicates significant differences in the means at *p* < 0.05 between each level in transgenic lines and WT plants, and ^**^, ^***^, and ^****^, in graphs, indicate significant differences in the means at *p* < 0.01, *p* < 0.001, and *p* < 0.0001, respectively.

To further explore the regulation pattern of the *ABI4* response pathway to low temperature, the expression pattern of related genes was identified. The results showed that under room temperature conditions, the expression of *COR413*, *ICE1*, and *OST1* was significantly lower in transgenic plants than in WT, while the opposite was true for *CBF1*, *CBF2*, *CBF3*, and *CBF4* (Figures 5A–G). The expression of *RAV1* did not exhibit significant differences between the transgenic plants and WT (Figure 5H). This suggested that *MtABI4* can affect the expression of the COR-CBF-ICE module and *OST1*, however, the effect on RAV1 was not significant.

After low-temperature treatment, there was a significant increase in the expression of *COR413*, *CBF4*, *ICE1*, and *OST1* in all lines, compared to those before low-temperature treatment (Figures 5A,E,F,G). Among them, the expression of *COR413* and *OST1* was significantly higher in transgenic plants than in WT, in contrast to before treatment. In addition, the expression of *ICE1* remained higher in WT, and the expression of *CBF4* remained higher in transgenic plants. The expression of the remaining genes, including *CBF1*, *CBF2*, and *RAV1* was down-regulated both in the transgenic plants and WT after low-temperature treatment (Figures 5B,C,H). Among them, the transgenic plants exhibited a significantly higher expression of *CBF1* and *CBF2* than WT, while the expression of *RAV1* was higher in WT. This indicated that the roles of the COR-CBF-ICE module and *OST1* and *RAV1* in the molecular response mechanisms induced by low temperature were somewhat altered under the influence of overexpressed *ABI4* genes.

### *MtABI4* directly interacts with MtABA2 protein and promotes the expression of the *ABA2* gene, which in turn increases abscisic acid content in transgenic plants

**FIGURE 6:**
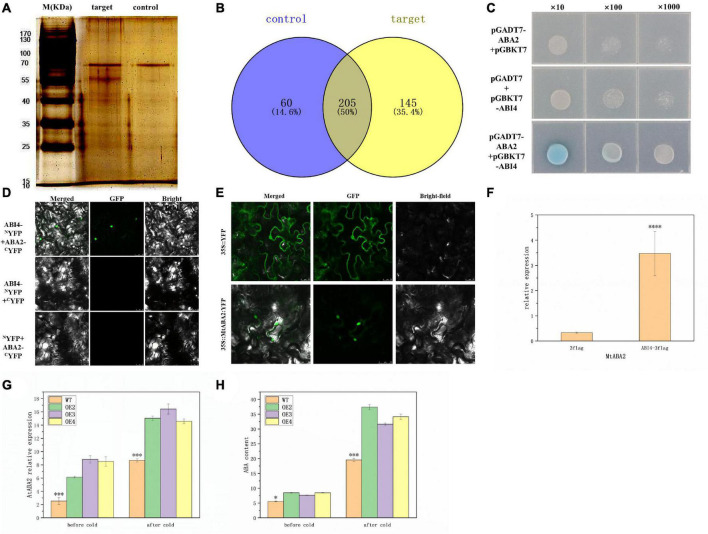
IP-MS, Y2H, BIFC, the expression level of ABA2, and ABA content. **(A)** Represents the results of the SDS-page electrophoretic. **(B)** Represents the intersection of the target and control mass spectrometry data. **(C)** Represents the growth of yeast in the Y2H analysis. **(D)** Represents the fluorescence observed in the BIFC analysis. **(E)** Represents the subcellular location of ABA2. **(F,G)** Represent ABA2 expression levels in *M. truncatula* callus, and in transgenic *Arabidopsis* before and after low-temperature treatment. **(H)** Represents the ABA level in the plant before and after the low-temperature treatment. *, in graphs, indicates significant differences in the means at *p* < 0.05 between each level in transgenic lines and WT plants, and ^***^, ^****^, in graphs, indicates significant differences in the means at *p* < 0.001 and *p* < 0.0001, respectively.

To screen for candidate proteins that might interact with MtABI4 protein, we purified ABI4-3flag fusion proteins transiently expressed in the wild-type callus of *Medicago* using flag magnetic beads. SDS-page electrophoretic analysis of the purified products showed that some candidate target proteins were obtained (Figure 6A). Mass spectrometry analysis showed that 145 target proteins were collected (Figure 6B), including a xanthoxin dehydrogenase gene (*ABA2*), considered a key role in abscisic acid biosynthesis (González-Guzmán et al., 2002). Further, the yeast two-hybrid assay revealed that only the mixture of yeast, containing pGADT7-*ABA2* and pGBKT7-*ABI4*, respectively, could be capable of normal growth and turn blue in SD medium lacking the four amino acids, while the other controls could not (Figure 6C). Bimolecular fluorescence complementation (BIFC) assay indicated that GFP fluorescence in nuclear was observed only in *Tobacco* cells co-transformed with *ABI4*-*^N^*YFP and *ABA2*-*^C^*YFP (Figure 6D). Together, these results demonstrated that MtABI4 could directly interact with MtABA2 protein *in vivo*. In addition, the ABA2 protein was also located in the nucleus (Figure 6E).

To further explore the regulatory pattern of *ABI4* on *ABA2*, the expression of *ABA2* in previously transfected callus and transgenic *Arabidopsis* was characterized by qRT-PCR. The results showed that the expression of *MtABA2* and *AtABA2* was up-regulated under the induction of *MtABI4* (Figures 6F,G). It suggested that over-expression of *MtABI4* could promote the expression of the *ABA2* gene both in *M. truncatula* callus and transgenic *Arabidopsis*. Meanwhile, the ABA content also increased in the transgenic *Arabidopsis* (Figure 6H). Previous studies showed that the main function of *ABA2* is to catalyze the conversion of xanthoxin to Abscisic Aldehyde which was confirmed as one of the last steps of the major ABA biosynthetic pathway (González-Guzmán et al., 2002). In conclusion, this was evidence that *MtABI4* could increase the ABA content in the transgenic *Arabidopsis* through the upregulated expression of *ABA2*. Furthermore, the overexpression of *MtABI4* also enhanced the expression of *ABA2* and improved the ABA biosynthesis of the plants, under low-temperature conditions (Figures 6G,H).

## Discussion

*ABI4* gene plays an integral role in plant growth and development and response to adversity. In this study, we found that the protein sequence of MtABI4 contains three elements and an AP2 structural domain, which is highly conserved with homologous proteins in other closely related legumes. This demonstrated that it was representative of investigating the function of the *MtABI4* gene from *Medicago truncatula*, a kind of model plant (Stacey et al., 2006), which could provide a reference for studying the function of homologous genes in other legumes. The MtABI4 protein was localized in the nucleus, and although this was inconsistent with our previous predictions, it was consistent with the localization of homologous proteins in *Arabidopsis* (Gregorio et al., 2014). This also confirms the accuracy of our results. It is important to note that YFP and GFP fluorescence have very close excitation light bands. To better avoid background interference, we chose the excitation band of GFP to observe the fluorescence of the YFP protein.

Analysis of the promoter sequence of the *ABI4* gene and hormone-induced expression analysis showed that the *ABI4* gene was able to respond to a variety of hormones, including IAA, 6-BA, GA, MeJA, SA, and ABA, which are thought to be associated with plant growth and development and stress response (Kazan, 2013; Colebrook et al., 2014). Several previous studies have suggested that *ABI4*, as a downstream of *DES1*, is involved in the ABA response in Arabidopsis (Zhou et al., 2021). ABA stabilizes the ABI4 protein, while GA promotes its degradation (Shu et al., 2016). This also coincides with our results that *MtABI4* was down-regulated under the treatment of GA, but up-regulated under the treatment of ABA. In addition, *ABI4* expression is induced by cytokinin (CTK) but inhibited by IAA (Shkolnik-Inbar and Bar-Zvi, 2010). Notably, our results indicated that although the expression of *ABI4* showed a similar decrease in the IAA treatment, it showed a decrease followed by an increase under the 6-BA treatment rather than a simple increase. This may be because the response of *ABI4* to CTK is phased in a temporal gradient. Moreover, SA and MeJA induced the expression of *PtrWRKY18* and *PtrWRKY35*, and overexpression of *PtrWRKY18* and *PtrWRKY35* down-regulated the expression of *ABI4* (Guo et al., 2020). It is reasonable to speculate that the inhibition of *ABI4* by SA and MeJA in this study may also be mediated by *WRKY18* and *WRKY35*. In summary, *ABI4* is widely involved in the molecular response mechanisms of manyp lant hormones and may play a pivotal role in hormone synergy.

In addition, we also discovered a low-temperature response element in the promoter region of *ABI4*. After the low-temperature treatment, the transgenic plants accumulated less anthocyanin and suffered less cell membrane damage. Synthesis and accumulation of anthocyanins are employed to contribute to plant resistance to low-temperature stress. For example, as previously reported, the cold tolerance of the *Hedera helix* increases as the total sugar and anthocyanin content of the leaves increases (Parker, 1962). Wild-type *Arabidopsis* plants are significantly more freeze-resistant than the four cold-sensitive mutants that do not synthesize anthocyanins (McKown et al., 1996). The northern ecotype *Populus trichocarpa* is more susceptible to surviving the winter than the southern ecotype due to its ability to accumulate more anthocyanins during the progressively shorter photoperiod (Chalker-Scott, 1999). This means that the transgenic plants in this study are insensitive to low temperatures and do not require the accumulation of large amounts of anthocyanin to respond dramatically to low temperatures, as the wild type does. Further, no significant differences in gene expression and enzyme activities of the four antioxidant enzymes appeared in transgenic plants and WT before the low-temperature treatment, different from the situation in low-temperature conditions. This suggests that *ABI4* may not act directly on these antioxidant enzyme genes, but indirectly affects their performance at low temperatures. After low-temperature treatment, overexpression of *ABI4* promoted the gene expression and enzyme activity of CAT and SOD but inhibited those of APX and POD. Numerous studies have suggested that gene expression and enzyme activity of SOD and CAT are positively correlated with plant cold tolerance (Gao et al., 2009; Zhang et al., 2018, 2021). This is also consistent with our results. In contrast, the significantly lower APX and POD enzyme activities in transgenic plants than in WT may be due to the insensitivity of the transgenic plants to low temperatures. This is worth exploring further in the future. In addition, the activities of antioxidant enzymes in plants are in chronological order, with a trend of increasing and then decreasing with the duration of low-temperature treatment (Ahmad et al., 2010). This may account for the significant decrease in antioxidant enzyme activity in both transgenic plants and WT after up to 5 days of cold treatment.

*ICE1* is the initial step in the ICE-CBF-COR signaling pathway, which plays a critical regulatory role in the low-temperature signaling pathway that involves multiple transcription factors (Stockinger et al., 1997). *TaICE1a* and *TaICE1d* were induced by cold treatment in wheat (Guo et al., 2019). Our results are also consistent with this, but the regulatory role of *MtABI4* on the expression of *ICE1* is more likely to be a repressor. And overexpression of *MdABI4* is capable of upregulating the *MdCBF1*, *MdCBF2*, *MdCBF3*, and *MdCOR47* in apple calli (An et al., 2022). Cold acclimation induces freezing tolerance in two chrysanthemums species by increasing *COR413* expression (Chen et al., 2014). Interestingly, except for those genes with the expression pattern consistent with previous results, *ABI4* acted as an inhibitor of *COR413* expression at room temperature but induced it at low temperature, whereas *CBF3* expression displayed the opposite trend. This “switch-like” regulation of *CBF3* and *COR413* by *ABI4* under low-temperature induction deserves further investigation. It has been shown that *COR* can only be activated to produce cold-regulated proteins only under certain conditions, such as low temperature and short sunlight, thus improving cold tolerance in plants (Jia et al., 2016). Under normal temperature, the HOS15-HD2C complex binds to the CORs promoter and represses expression by inducing CORs chromatin deacetylation. HOS15 enhances the binding of CBFs to the CORs promoter by activating the E3 ligase CUL4 (CULLIN4) for ubiquitinated degradation of HD2C under low-temperature stress (Park et al., 2015). It is reasonable to assume that *ABI4* may also play a similar role to that of *HOS15* in the regulation of *COR413* and *CBF3*. Apart from this, several studies have suggested that *RAV1* and *OST1* are also actively involved in adaption to cold stress in plants. For instance, *VaRAV1* may promote plant cold tolerance in grapevine cells via improving cell membrane stability and inducing the expression of genes involved in plant cell wall composition (Ren et al., 2021). At low temperatures, OST1 can be activated to phosphorylate ICE1, leading to its higher stability and transcriptional activity (Ding et al., 2015). In our results, their regulatory effects by *ABI4* also displayed a similar pattern to that of *CBF3* and *COR413*, which may be a new perspective to explore how *ABI4* and these cold-inducible genes are mutually regulated and act together in the process of plant low-temperature response.

*ABA2* involved in the final step of the ABA biosynthetic pathway plays a vital role in the regulation of endogenous ABA levels in plants (González-Guzmán et al., 2002). It has been shown that the Aba2-2 mutant has lower levels of endogenous ABA than the wild type (Nambara et al., 1998). This exactly complements our results that the elevation of *ABA2* expression levels caused by *ABI4* overexpression was accompanied by an increase in ABA content. ABA is an important signaling factor for stressful low temperatures and has a protective function for the microtubule structure of cells, and plants under low-temperature stress accumulate ABA in large quantities and rapidly to perform a protective function (Lang et al., 1994). The foliar application of exogenous ABA increased the endogenous ABA content of the leaves and improved the tolerance of the crop to low temperatures (Hongtao et al., 2017). Under low-temperature conditions, exogenous ABA can increase the content of proline, soluble sugar, and soluble protein in rice leaves, effectively increase SOD and CAT activities, reduce Malondialdehyde (MDA) accumulation and enhance cold tolerance of rice. Another more direct strategy adopted in our study was to promote the expression of *ABA2* and enhance the accumulation of endogenous ABA, through the overexpression of *ABI4*, thus improving the cold tolerance of plants.

## Conclusion

**FIGURE 7:**
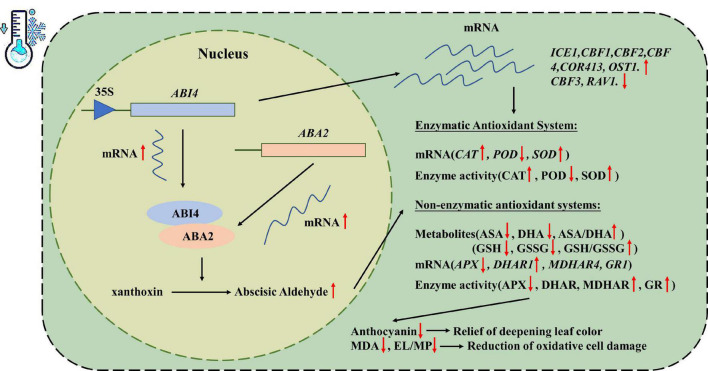
Putative regulatory mechanisms of *MtABI4*.

MtABI4 protein was localized in the cell nucleus and could be induced by multiple hormones, including IAA, GA, MeJA, SA, 6-BA, and ABA. Moreover, MtABI4 could directly interact with MtABA2 protein and promotes the expression of the *ABA2* gene, which in turn increases abscisic acid content in transgenic plants. At low temperatures, the high accumulation of ABA in transgenic plants led to increased SOD, CAT, MDHAR and GR activity, a lower MDA level, a higher ASA/DHA ratio and GSH/GSSG ratio, and reduced anthocyanin content, thus providing some protection against low-temperature damage and leaf color deepening in plants. In addition, overexpression of *ABI4* also induced the expression of members of the ICEs-CBFs-CORs low-temperature response module. These factors combined to improve the cold tolerance of *ABI4* overexpression plants (Figure 7). This study provides a new insight into the mechanisms of ABA-mediated regulation of plant cold tolerance and offers a molecular theoretical basis for plant cold tolerance improvement.

## Materials and methods

### Plant materials, growth conditions, and treatments

The seeds of *M. truncatula* (R108), *Arabidopsis thaliana*, and *Nicotiana benthamiana*, and plant expression vector 3302Y, 3302-3flag, and *Agrobacterium tumefaciens* EHA105 strain used in this study were preserved in the laboratory of turfgrass science and management of Beijing forestry university. These seeds were vernalized for three days at 4°C and then germinated on the sterile filter paper for about one week. The seedlings were transferred to a vegetative soil matrix (peat: vermiculite = 1:1). *Medicago* and *Nicotiana* were cultivated in the greenhouse under the condition of a 16 h light/8 h dark cycle at 26°C/24°C, and *Arabidopsis* were under a 16 h light/8 h dark cycle at 23°C/21°C. After two months of cultivation, *Medicago* with similar growth was selected and sprayed with 10 μmol/L IAA, 10 μmol/L 6-BA, 10 μmol/L GA3, 10 μmol/L ABA, 10 μmol/L MeJA, and 0.5 mmol/L SA (Guo et al., 2020), respectively, and the plant tissues were collected at 0, 1, 3, 6, 9, and 12 h for temporal expression analysis.

### Gene cloning and bioinformatics analysis

Total RNA was extracted from well-grown leaves of *M. truncatula*, and then reverse transcribed into cDNA by using the PrimeScript RT reagent Kit with gDNA Eraser from TAKARA. Using cDNA as a template and ABI4-CLONE-F, and ABI4-CLONE-R (Supplementary Table 1) as primers, the CDS of this gene was obtained by RT-PCR. And then it was attached to the pMD19-T clone vector from TAKARA, named as ABI4-pMD19-T vector, and was preserved in DH5α *E. coli* from TAKARA for further analysis.

DNAMAN 6.0 was used to analyze the sequence of *MtABI4* and predict the encoded amino acid sequence. Blast homology comparison was performed on the NCBI website. ABI4 homologous proteins in different species were mapped into evolutionary trees by MEGA 5.0. MEME website^1^ was used for motif analysis. The results above were visualized via TBtools (Chen Chengjie, Guangzhou, Guangdong, China). SignalP 4.1 Server website^2^ and Softberry website^3^ were used to predict signal peptide cleavage sites and subcellular localization, respectively.

### Subcellular localization and spatio-temporal expression analysis

Using the ABI4-pMD19-T vector as a template and ABI4-3302y-F, ABI4-3302y-R (Supplementary Table 1) as primers, the 35S:*MtABI4*:*YFP* expression vector was constructed by PCR and was turned into EHA105 *A. tumefaciens* for analysis of subcellular localization. The qualified bacterial solution above was injected into the young tender leaves of *Nicotiana benthamiana*. After 48 h of dark culture, the infected leaves were observed with LEICA SP-8 confocal microscope to determine the localization of the ABI4 protein. The localization of ABA2 protein was analyzed by the same methods.

The root, stem, leaf, flower, seeds, and pods of wild-type *M. truncatula* were collected and stored at −80°C after quick freezing with liquid nitrogen for further analysis of spatial differential expression. Total RNA of these tissues was extracted and then transcribed into cDNA as a template for quantitative fluorescence detection. Using ABI4-RT-F, ABI4-RT-R (Supplementary Table 1) as primers and the *MtActin* gene as the internal reference, real-time fluorescence quantitative analysis of the *MtABI4* gene was completed by the CFX Connect RT-PCR system. The reaction conditions were as follows: 95°C for 10 min;95°C 15 s, 60°C 1 min, 40 cycles. And the relative expression of *ABI4* was determined by the 2^–ΔΔCT^ method (Guo et al., 2020). All samples were processed with three biological replicates. Temporal expression analysis of *MtABI4* adopted the same method mentioned above. The primers used were shown in the Supplementary Table 1.

### Generation and identification of transgenic plants

The 35S:*MtABI4*:*YFP* expression vector was transformed into *Arabidopsis* plants by the floral dip method (Guo et al., 2020). Positive transgenic plants were screened by spraying 60 mg⋅L^–1^ glyphosate and then identified by PCR and RT-PCR. T3 generation transgenic lines were cultivated for further treatment and analysis. See Supplementary Table 1 for the primers used.

### Chlorophyll content

The chlorophyll content was determined using 95% ethanol as described in the previous report (Teng et al., 2021). Total chlorophyll, Chl a, Chl b, and carotenoid contents were calculated based on the absorbance recorded. All the samples were replicated three times.

### Anthocyanin content

The anthocyanin content was determined using 10 mL 1% methanol hydrochloride solution (pH = 0–2). After shaking at 30°C for 5 h, the absorbance at 530 nm was measured.

### Electrolyte leakage and cell membrane permeability

0.1 g plant sample in 25 mL distilled water was shaken for 24 h at room temperature. The electrolyte leakage of supernatant (EL1) was measured. After boiling water bath for 30 min, the electrolyte leakage of supernatant (EL2) was measured. The electrolyte leakage of distilled water (EL0) with the same shaking and boiling was also measured as a control. The electrolyte leakage of samples was calculated as EL1/EL2, and the cell membrane permeability was calculated as (EL1- EL0)/(EL2- EL0).

### Antioxidant enzyme activity and malondialdehyde content

SOD activity was measured by photoreduction with nitrogen blue tetrazolium (NBT). 3.4 mL of the reaction mix and 0.3 mL of 20 μmol L^–1^ riboflavin solution were added to 100 μL of the enzyme solution and then mix well, with no enzyme solution shaded with tin foil as control. The tubes were placed under a 4,000 l× lamp for 20–30 min and the OD values were measured at 560 nm.

POD activity was measured by guaiacol colorimetric assay. The reaction solution, containing 1 mL of phosphate buffer with 50 mmol L^–1^ pH 7.0, 0.95 mL of 0.2%guaiacol, 1 mL of 0.3% H_2_O_2_, and 50 μL of enzyme solution was added to a spectrophotometer cup (1 cm radius) and then mix well with no enzyme solution as a control. The kinetic OD values were measured at 470 nm in one minute. The Time interval was 5 s.

CAT activity was measured by UV spectrophotometry. 2.9 mL of the reaction solution, containing 20 mmol L^–1^ H_2_O_2_ solutions was added to 100 μL of enzyme solution and mix well. Distilled water was used as a blank control, the kinetic OD values were measured at 240 nm in one minute. The time interval was 5 s.

APX activity was measured by ascorbic acid colorimetric assay. The reaction solution, containing 2.82 mL of phosphate buffer with 50 mmol L^–1^ pH 7.0, 30 mL of 50 mmol L^–1^ ascorbic acid, 30 μL of 10 mmol L^–1^ EDTA, and 100 μL of enzyme solution was added to a spectrophotometer cup (1 cm radius) and then mix well with no enzyme solution as a control. The kinetic OD values were measured at 290 nm in one minute. The time interval was 5 s.

MDA content was measured by the thiobarbituric acid (TBA) reduction method. 1 mL of the enzyme solution, mixed with 2 mL TBA in a 5 mL centrifugal tube was boiled for 30 min. The tubes were then rapidly transferred to an ice bath for cooling to abort the reaction. After centrifugation at 10,000 *g* for 10 min, the supernatant was taken and the absorbance values at 600 and 532 nm were measured. All the samples above were replicated three times.

### Determination of indicators related to non-enzymatic antioxidant systems

The content of ASA, DHA, GSH, and GSSG and the activities of DHAR, MDHAR, and GR was measured by the corresponding kits (No.G0201F, G0202F, G0206F, G0207F, G0212F, G0213F, and G0209F in turn) from GERUISI-BIO (Suzhou, China).

### Abscisic acid content

ABA content were determined by high-performance liquid chromatography (Lu et al., 2010).

### Immunoprecipitation, coupled with mass spectrometry

The entire experimental procedure for the Immunoprecipitation-mass spectrometry (IP-MS) analysis was referenced from the previous method with some modifications (Cao and Yan, 2016). Wild-type callus of *M. truncatula* was obtained via plant tissue culture. The ABI4-3302-3FLAG expression vector, constructed in a similar way to the aforementioned vectors, was transformed into callus by an Agrobacterium-mediated method for transient expression of ABI4 protein. In addition, the 3302-3FLAG expression vector was also transformed into the callus as a control.

After three days of dark incubation, the total protein of the callus was extracted by grinding in PBS buffer (pH = 7.4). The protein samples were purified using BeyoMag™ Anti-Flag Magnetic Beads (Beyotime, China). The end products obtained by elution were subjected to electrophoretic analysis using 10% SDS-PAGE gel. When bands were observed on the silver-stained gel treated with the Fast Silver Stain Kit (Beyotime, China), the gel block was sent to Bio for mass spectrometry analysis (Biomarker, China). The candidate proteins that interacted with the target protein were obtained by subtracting the protein in the control group from the experimental group results.

### Yeast two-hybrid

pGADT7-*ABA2* and pGBKT7-*ABI4* expression vectors were constructed by a similar method above and transformed into Y187 and yeast two-hybrid (Y2H) yeast strains in turn. The mixture of these two kinds of yeast was cultivated in SD solid medium lacking Leu, Trp, His, and Ade, supplemented with 40 μg ml^–1^ X-α-gal and 125 ng ml^–1^ Aureobasidin A, for 3–5 days at 29.5°C. Moreover, they matched with the yeast strains containing pGADT7or pGBKT7 empty vector and were cultivated under the same conditions as controls.

### Bimolecular fluorescence complementation

*ABI4*-*^N^*YFP and *ABA2*-*^C^*YFP expression vectors were constructed by a method similar to those described above and transformed into *Agrobacterium tumefaciens* EHA105 strain. These fluids were injected into tobacco leaves by a method similar to subcellular localization. The mixture of strains containing *ABI4*-*^N^*YFP and *ABA2*-*^C^*YFP was the treatment group and these two strains were mixed with those containing the *^N^*YFP or *^C^*YFP empty vectors, separately, as controls. GFP fluorescence in *tobacco* cells was observed with LEICA SP-8 confocal microscope, after 2 days of dark incubation.

### Statistical analysis

Data were analyzed by SPSS 15.0, Excel 2020, GraphPad Prism 9.0, and Origin 2022. All data were presented as the mean ± SD (*n* = 3).

## Data availability statement

The original contributions presented in this study are included in the article/Supplementary material, further inquiries can be directed to the corresponding author.

## Author contributions

YL proposed ideas and designed the experiments and wrote the manuscript. YL, SL, and MW completed the experiments. YL, TG, and KT analyzed the experimental data. DD, ZL, and YC proposed key opinions that cannot be ignored. LH and CJ played a guiding role in the whole work process. All authors read and reached an agreement with the final manuscript.

## Footnotes

1
https://meme-suite.org/meme/tools/meme

2
http://www.cbs.dtu.dk/services/SignalP/

3
http://www.softberry.com/

## Supplementary material

Supplementary Figure 1 Bioinformatic analysis of *MtABI4*.
