# Supplementary figures and images for: Overexpression of abscisic acid-insensitive gene ABI4 from Medicago truncatula, which could interact with ABA2, improved plant cold tolerance mediated by ABA signaling
Source: Front Plant Sci. 2022 Sep 23;13:982715. doi: 10.3389/fpls.2022.982715 (PMC9545351; doi:10.3389/fpls.2022.982715)

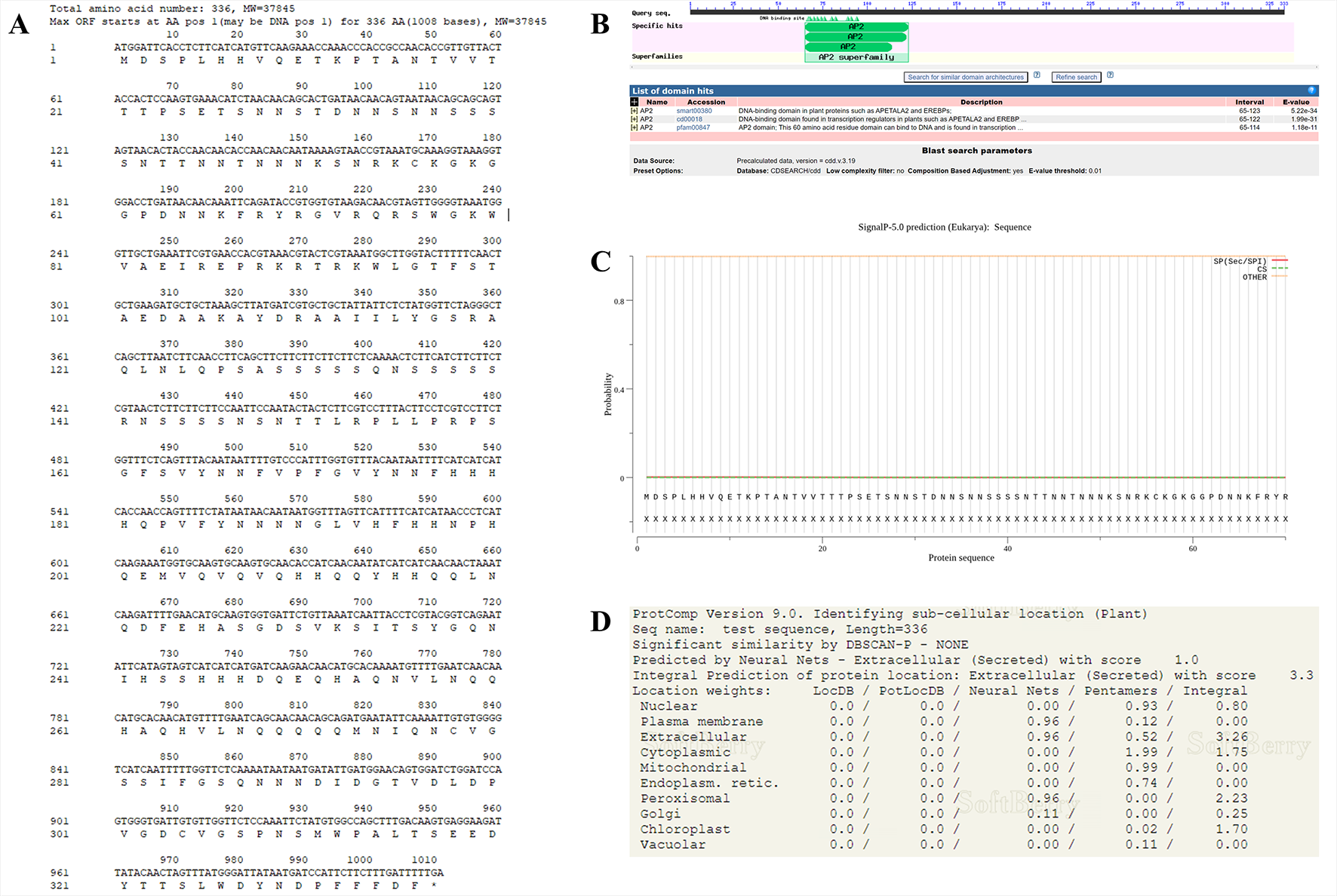

Supplement: Supplementary Figure 1 — Bioinformatic analysis of MtABI4. [file Image_1.TIF]
